# Mersaquinone, A New Tetracene Derivative from the Marine-Derived *Streptomyces* sp. EG1 Exhibiting Activity against Methicillin-Resistant *Staphylococcus aureus* (MRSA)

**DOI:** 10.3390/antibiotics9050252

**Published:** 2020-05-14

**Authors:** Min Cheol Kim, Reiko Cullum, Ali M. S. Hebishy, Hala A. Mohamed, Ahmed H. I. Faraag, Nehad M. Salah, Mohamed S. Abdelfattah, William Fenical

**Affiliations:** 1 Center for Marine Biotechnology and Biomedicine, Scripps Institution of Oceanography, University of California, San Diego, La Jolla, CA 92093, USA; mck008@ucsd.edu (M.C.K.); rcullum@ucsd.edu (R.C.); nehad.mo.salah@std.pharma.cu.edu.eg (N.M.S.); 2 Natural Products Research Unit, Chemistry Department, Faculty of Science, Helwan University, Cairo 11795, Egypt; ashebishy@gmail.com (A.M.S.H.); Halaasklany89@gmail.com (H.A.M.); 3 Botany and Microbiology Department, Faculty of Science, Helwan University, Cairo 11795, Egypt; professor.ahmed85@googlemail.com

**Keywords:** MRSA, marine actinomycetes, *Streptomyces* sp., anthraquinone, spectroscopy

## Abstract

New antibiotics are desperately needed to overcome the societal challenges being encountered with methicillin-resistant *Staphylococcus aureus* (MRSA). In this study, a new tetracene derivative, named Mersaquinone (**1**), and the known Tetracenomycin D (**2**), Resistoflavin (**3**) and Resistomycin (**4**) have been isolated from the organic extract of the marine *Streptomyces* sp. EG1. The strain was isolated from a sediment sample collected from the North Coast of the Mediterranean Sea of Egypt. The chemical structure of Mersaquinone (**1**) was assigned based upon data from a diversity of spectroscopic techniques including HRESIMS, IR, 1D and 2D NMR measurements. Mersaquinone (**1**) showed antibacterial activity against methicillin-resistant *Staphylococcus aureus* with a minimum inhibitory concentration of 3.36 μg/mL.

## 1. Introduction

Methicillin-resistant *Staphylococcus aureus* (MRSA) is an infectious Gram-positive bacterium that is widely distributed worldwide in hospitals, convalescent homes and community settings [1]. It causes serious infections to the skin and soft tissues and also systemic infections that are responsible for significant mortalities [2,3,4]. MRSA is multidrug resistant and has evaded successful control [5]. Consequently, discovery of new antibiotics is urgently needed to control MRSA infections.

Marine bacteria have been shown to produce compounds that inhibit the growth of methicillin-resistant *Staphylococcus aureus* [6,7,8,9]. Among these bacteria, actinomycetes appear to be the most prolific producers [10,11]. The marine-derived actinomycetes are known to produce compounds of unique structures with antimicrobial activities [12,13,14]. As per the literature, marine actinomycetes are widely distributed in seawater, sea sand, and deep-sea sediments [15,16,17]. The Mediterranean Sea, which has received relatively little examination, is one of the most important marine ecosystems in Egypt and considered as a region of high biodiversity [18,19]. The Mediterranean Sea is known to possess unique marine habitats, including those dominated by macroalgae, seagrasses, invertebrates, seabirds and different types of microbes [19]. Only a few studies have been carried out to isolate natural compounds from marine-derived actinomycetes of the Mediterranean Sea [20,21]. This paper reports the isolation and structure elucidation of a new tetracene derivative (**1**) from the culture extract of the marine-derived *Streptomyces* sp. EG1.

## 2. Results and Discussion

**Figure 1:**
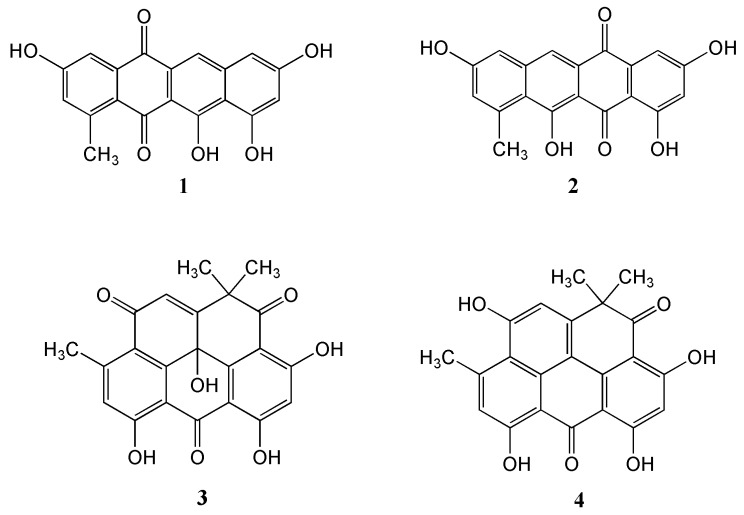
Structures of the isolated compounds **1**–**4**.

The marine-derived *Streptomyces* sp. EG1 was isolated from a sediment sample collected near Mersa Matruh city along the Mediterranean coast of Egypt. Cultivation of the strain on a Waksman medium at 28 °C for 7 days followed by extraction, evaporation and chromatographic purification of the organic extract led to the isolation of one new compound Mersaquinone (**1**) as well as the known Tetracenomycin D (**2**) [22], Resistoflavin (**3**) [23] and Resistomycin (**4**) [24] (Figure 1).

**Figure 2:**
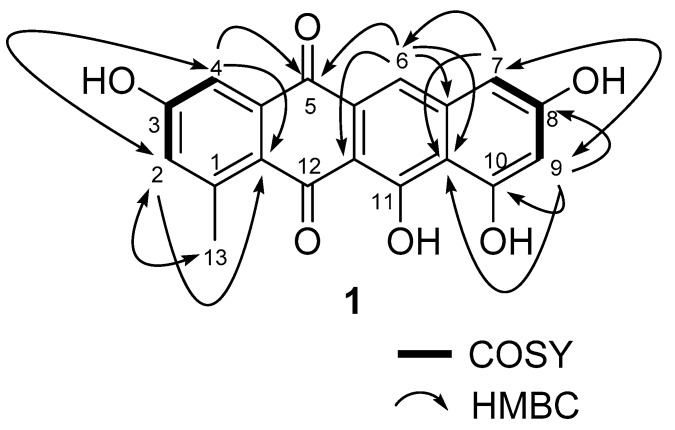
Key COSY and HMBC correlations for compound **1**.

Mersaquinone (**1**) was isolated as a red amorphous powder and its molecular formula was assigned as C_19_H_12_O_6_ based on HR-ESI-MS data ([M+H]^+^ at *m/z* 337.0714, Figure S1). The UV spectrum of **1** measured in MeOH exhibited absorption bands at 218, 277, 308, 350, 480, 515 and 550 nm, indicating the presence of an anthraquinone moiety [25]. The IR spectrum of **1** showed absorption bands for OH (3475 cm^−1^) and carbonyl groups (1685 cm^−1^). The ^1^H NMR spectrum (Figure S2) showed five aromatic methine signals at *δ*_H_ 7.75 (s), 7.45 (br d, *J* = 2.2 Hz), 7.00 (br d, *J* = 2.2 Hz), 6.84 (br d, *J* = 1.8 Hz), and 6.51 (br d, *J* = 1.8 Hz) and one methyl group at *δ*_H_ 2.70. The ^13^C NMR spectrum (Figure S3), and gHSQC NMR spectrum (Figure S4) of **1** displayed 19 carbon signals composed of two carbonyl carbon signals (*δ*_C_ 186.4, and 181.8), 11 quaternary *sp^2^* carbon signals (*δ*_C_ 162.2, 162.0, 158.0, 157.8, 137.7, 145.2, 138.9, 122.6, 109.7, 106.1, and 128.0), five aromatic methine carbon signals (*δ*_C_ 124.8, 120.2, 112.2, 106.4, and 104.7), and one methyl signal (*δ*_C_ 23.9). The ^1^H-^1^H COSY NMR spectrum (Figure S5) displayed correlations of δ_H_ 7.45 (H-4) and 7.00 (H-2), and of δ_H_ 6.84 (H-7) and 6.51 (H-9), which showed long-range *meta* spin-spin coupling constants in the ^1^H NMR spectrum. In the HMBC spectrum (Figure S6), the two *meta* coupled protons signals showed long-range HMBC correlations from H-2 to C-4, C-13 and C-12a, H-4 to C-2, C-5, and C-12a, H-7 to C-6, C-9, and C-10a, and H-9 to C-7, C-8, C-10, and C-10a. Additionally, the aromatic methine proton H-6 correlated to C-5, C-6a, C-10a, and C-11a (Figure 2). These results indicated the presence of a 3,8,10,11-tetrahydroxy-1-methyltetracene-5,12-dione ring system. Based on the above spectroscopic data, the full planar structure of Mersaquinone (**1**), was confirmed as shown in Figure 1. The known compounds **2**–**4** were identified by comprehensive NMR and MS analysis (Table S1 and Figures S7–S17).

The antibacterial activity of Mersaquinone (**1**) was evaluated against methicillin-resistant *Staphylococcus aureus* (MRSA) strain TCH1516. The carbon skeleton of tetracenomycin derivatives has been reported to show anti-methicillin-resistant *Staphylococcus aureus* (MRSA) activities [26,27]. Our results showed that **1** had anti-MRSA activity with an MIC value of 3.36 μg/mL. Ciprofloxacin HCl hydrate was used as a positive control and showed antibacterial activity with an MIC value of 0.93 μM. As per literature, Napyradiomycins A80915 and A80915B with naphthoquinone moiety, isolated from the marine-derived *Streptomyces* sp. CNQ-525, exhibited potent antibacterial activity against contemporary MRSA strains [28]. Both Napyradiomycin derivatives displayed MIC values in the range of 1–3 μg/mL. Balachandran et al. [29] also reported that 2-hydroxy-9,10-anthraquinone from *Streptomyces olivochromogenes* showed anti-MRSA activity with MIC value of 50 μg/mL.

## 3. Materials and Methods

### 3.1. General Experimental Procedures

The UV spectra were measured with a Beckman Coulter DU800 spectrophotometer with a path length of 1 cm, and IR spectra were acquired on a JASCO FTIR-4100 spectrometer. The 1D and 2D NMR spectroscopic data were obtained on a JEOL 500 NMR spectrometer. The values of the chemical shifts are described in ppm and coupling constants are reported in Hz. The high-resolution ESI-TOF mass spectral data were recorded on an Agilent 6530 Accurate-Mass Q-TOF. The mass spectrometer was coupled to an Agilent 1260 LC system with a Phenomenex Luna C18 column (4.6 × 100 mm, 5 µm, flow rate 0.7 mL/min). Preparative HPLC separations were performed using a Shimadzu SCL-10A with a Shimadzu SPD-M10A UV/Vis detector and a reversed-phase C18 column (Phenomenex Luna, 10.0 × 250 mm, 5 µm) at a flow rate of 3.0 mL/min.

### 3.2. Isolation and Identification of Streptomyces sp.EG1

A sediment sample was collected from Mersa Matruh city on the North Coast of the Mediterranean Sea of Egypt. Briefly, one gram of wet sediment was dispersed in 9 mL of sterilized water and vortexed for 3 min. The sample was subjected to heat treatment at 60 °C for 15 min to remove non-sporulating bacteria. A serial dilution (10^−1^, 10^−2^ and 10^−3^) of the suspension with sterilized seawater was carried and an aliquot (100 μL) was spread on starch-casein agar plate (starch 10 g/L, KNO_3_ 2 g/L, casein 0.3 g/L, NaCl 2 g/L, K_2_HPO_4_ 2 g/L, MgSO_4_ 7 H_2_O 0.05 g/L, CaCO_3_ 0.02 g/L, FeSO_4_ 7 H_2_O 0.01 g/L, agar 18 g/L, 50% seawater and 50% deionized water) [30,31]. Cycloheximide (50 μg/mL) and nalidixic acid (75 μg/mL) were added to the media as antifungal and antibacterial agents, respectively. The plates were kept at 28 °C for 15 days until colonies appeared. Colonies that produced an orange pigment were selected and purified by streaking on Waksman seawater agar plates. The strain, our voucher EG1, was identified as a *Streptomyces* sp. by 16S rRNA gene sequence analysis (GenBank accession no. MT186138). The closest matching strain was *Streptomyces griseorubens* strain IMB16-121 (99.85% identity; Sequence ID: MG190723.1).

### 3.3. Cultivation, and Extraction of Streptomyces sp. EG1

The strain was cultivated in 1 L scale using a Waksman medium (WM). A seed culture was first prepared by adding a piece of an agar plate with a growth colony into a 250 mL Erlenmeyer flask containing 25 mL of a Waksman medium (Glucose (20 g/1 L), peptone (5 g/1 L), meat extract (5 g/1 L), yeast extract (3 g/1 L), CaCO_3_ (3 g/1 L), NaCl (5 g/1 L), 50% seawater, 50% deionized water) and then cultivated on a rotary shaker at 120 rpm, 28 °C for 5 days as a seed culture. The seed culture (20 mL) was then added to a 2.8 L Fernbach flask containing 1 L WM. After 7 days of cultivation, 20 g of XAD-7 resin was added to the broth. The resin was collected and extracted with acetone, and the solvent was removed under vacuum. The remaining solution was then extracted with ethyl acetate, and the ethyl acetate layer was collected and evaporated under reduced pressure to yield 0.59 g of organic extract.

### 3.4. Isolation of Compounds

The organic extract (0.59 g) was subjected to silica gel vacuum flash chromatography (Merck Type 60, 3 × 50 mm), using stepwise elution with CH_2_Cl_2_ and MeOH (100:0, 100:1, 50:1, 10:1, 5:1, 1:1, and 0:100; each 40 mL) to afford seven fractions. Fractions **4** (232 mg) and **5** (63 mg) were further purified by repeated C-18 reverse-phase HPLC (Phenomenex Luna C-18 column, 10 × 250 mm, 5 µm column; 3 mL/min flow rate, UV detection at 254 nm) with 50% to 100% MeCN/H_2_O over 25 min with elution thereafter isocratic with the same solvent for 5 min to yield four pure compounds: compound **1** (3.4 mg, *t*_R_ 15.7), **2** (5.2 mg, *t*_R_ 17.2), **3** (6.6 mg, *t*_R_ 21.8), and **4** (9.6 mg, *t*_R_ 28.7).

### 3.5. Spectral Data of Mersaquinone

Red amorphous powder. IR (ZnSe) ν_max_ 3457, 1685, 1592, 1438, 1359, 1326, 1209, 1193 cm^−1^. UV (MeOH) λ_max_ (log ε) 218 sh (3.44), 277 (3.68), 308 sh (3.34), 350 sh (3.95), 480 sh (3.09), 515 (3.20), 550 sh (3.04). ^1^H NMR (500 MHz, DMSO-*d_6_*) δ_H_: 7.75 (1H, s, 6-H), 7.45 (1H, d, *J* = 2.2 Hz, H-4), 7.00 (1H, d, *J* = 2.2 Hz, H-2), 6.84 (1H, d, *J* = 1.8 Hz, H-7), 6.51 (1H, d, *J* = 1.8 Hz, H-9), 2.70 (3H, s, 13-CH_3_). ^13^C NMR (125 MHz, DMSO-*d*_6_) δ_C_: 185.4 (Cq-12), 181.8 (Cq-5), 162.2 (Cq-8), 162.0 (Cq-3), 158.0 (Cq-11), 157.8 (Cq-10), 145.2 (Cq-1), 138.8 (Cq-6a), 137.7 (Cq-4a), 128.0 (Cq-5a), 124.8 (CH-2), 122.6 (Cq-12a), 120.2 (CH-6), 112.2 (CH-4), 109.7 (Cq-10a), 106.4 (CH-7), 106.1 (Cq-11a), 104.7 (CH-9), 23.9 (CH_3_-13). HR-ESI-TOF-MS: *m/z* 337.0714 [M+H]^+^ (calcd. For C_19_H_13_O_6_, 337.0712).

### 3.6. Antibacterial Testing

The methicillin-resistant *Staphylococcus aureus* (MRSA) strain TCH1516 was used in our study. The minimum inhibitory concentration (MIC) for Mersaquinone (**1**) was evaluated by the broth microdilution method according to CLSI guidelines [32]. Briefly, a few colonies of a pure culture of TCH1516 on agar were put into cation adjusted Mueller–Hinton broth (CAMHB). The inoculum was diluted in CAMHB broth to give a final organism density of 1×10^5^ cfu/mL. Ten-fold serial dilutions of Mersaquinone (**1**) were dispensed in a microtiter plate as well as the inoculum. The overall volume in each well was 180 μL. The plate was incubated at 37 °C in air for 20 h, then the optical density (OD) at 650 nm was read using a plate reader (EmaxPrecision Microplate Reader by Molecular Devices). Ciprofloxacin HCl hydrate was used as a positive control. Negative control and quality control showing growth of pathogen were carried out in replicate analyses. The MIC assay was carried out using molarity concentrations at 10 fold dilutions. The MIC (obvious no growth) was confirmed in one of the 10-fold dilutions at 10 micro M, which is equal to 3.36 mcg/mL.

## 4. Conclusions

A new tetracene derivative, Mersaquinone (**1**), was isolated from the culture broth of the marine-derived *Streptomyces* sp. strain EG1. Mersaquinone (**1**) shows moderate antibacterial activity against methicillin-resistant *Staphylococcus aureus* (MRSA). Our study revealed that sediments of the Mediterranean Sea are a good source of actinomycetes, which may produce new compounds with promising biological activities.

## Supplementary Materials

The following are available online at https://www.mdpi.com/2079-6382/9/5/252/s1, Figure S1: chromatographic data and HR-ESI-MS data, as well as 1D and 2D data for mersaquinone (**1**), and 1D and 2D NMR data for compounds 2-4. Table S1: NMR spectroscopic data for compounds **1** and **2**. Figure S2. 1H NMR data of mersaquinone (1), 500 MHz, DMSO-d6; Figure S3. 13C NMR data of mersaquinone (1), 125 MHz, DMSO-d6.; Figure S4. gHSQC NMR data of mersaquinone (1), 500 MHz, DMSO-d6; Figure S5. 1H-1H COSY NMR data of mersaquinone (1), 500 MHz, DMSO-d6; Figure S6. gHMBC NMR data of mersaquinone (1), 500 MHz, DMSO-d6; Figure S7. 1H NMR data of compound 2, 500 MHz, DMSO-d6; Figure S8. 13C NMR data of compound 2, 125 MHz, DMSO-d6; Figure S9. 1H-1H COSY NMR data of compound 2, 500 MHz, DMSO-d6; Figure S10. gHSQC NMR data of compound 2, 500 MHz, DMSO-d6; Figure S11. gHMBC NMR data of compound 2, 500 MHz, DMSO-d6; Figure S12. 1H NMR data of compound 3, 500 MHz, DMSO-d6; Figure S13. 13C NMR data of compound 3, 125 MHz, DMSO-d6; Figure S14. gHSQC NMR data of compound 3, 500 MHz, DMSO-d6; Figure S15. gHMBC NMR data of compound 3, 500 MHz, DMSO-d6; Figure S16. 1H NMR data of compound 4, 500 MHz, DMSO-d6; Figure S17. 13C NMR data of compound 4, 125 MHz, DMSO-d6.
