# Supplementary material for: Mersaquinone, A New Tetracene Derivative from the Marine-Derived Streptomyces sp. EG1 Exhibiting Activity against Methicillin-Resistant Staphylococcus aureus (MRSA)
Source: Antibiotics (Basel). 2020 May 14;9(5):252. doi: 10.3390/antibiotics9050252 (PMC7277363; doi:10.3390/antibiotics9050252)

Supporting Information

Mersaquinone, a New Tetracene Derivative from the Marine-Derived *Streptomyces* sp. EG1 Exhibiting Activity Against Methicillin-Resistant *Staphylococcus aureus* (MRSA)

Min Cheol Kim[1], Reiko Cullum[1], Ali M. S. Hebishy[2], Hala A. Mohamed[2], Ahmed H. I. Faraag[3] Nehad M. Salah[1], Mohamed S. Abdelfattah[2] *, and William Fenical[1]*

Figure S1. Chromatographic data and HR-ESI_MS data for mersaquinone (**1**).

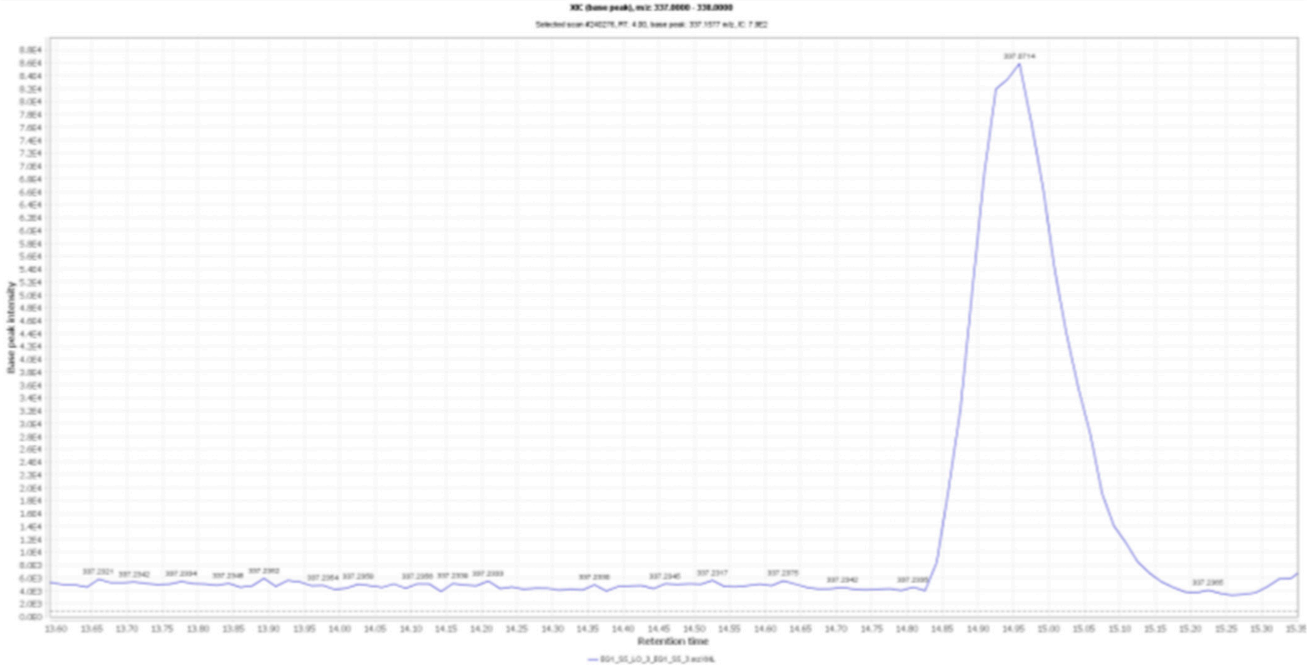

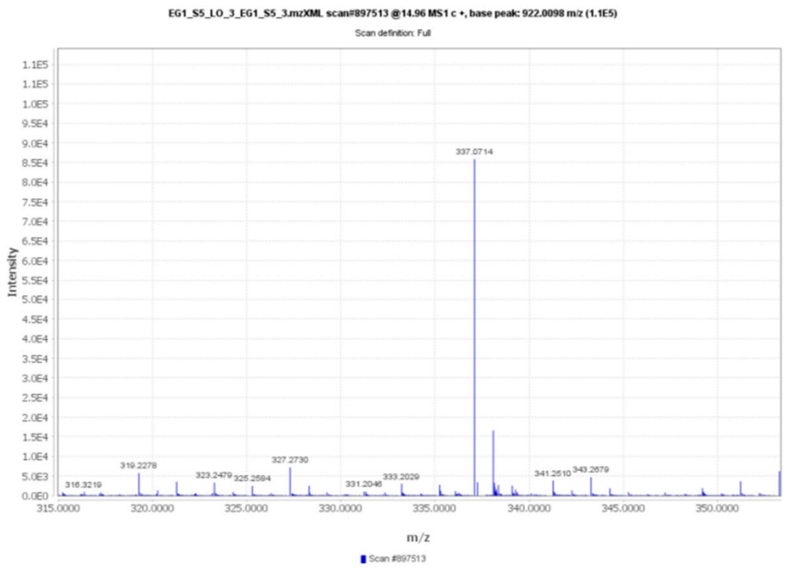
EG1_S5_LO_3_EG1_S5_3.mzXML scan#897513 @14.96 MS1 c +, base peak: 922.0098 m/z (1.1E5)
Scan definition: Full
Intensity
m/z
337.0714
319.2278
323.2479
325.2584
327.2730
331.2046
333.2029
341.2510
343.2679
316.3219
Scan #897513

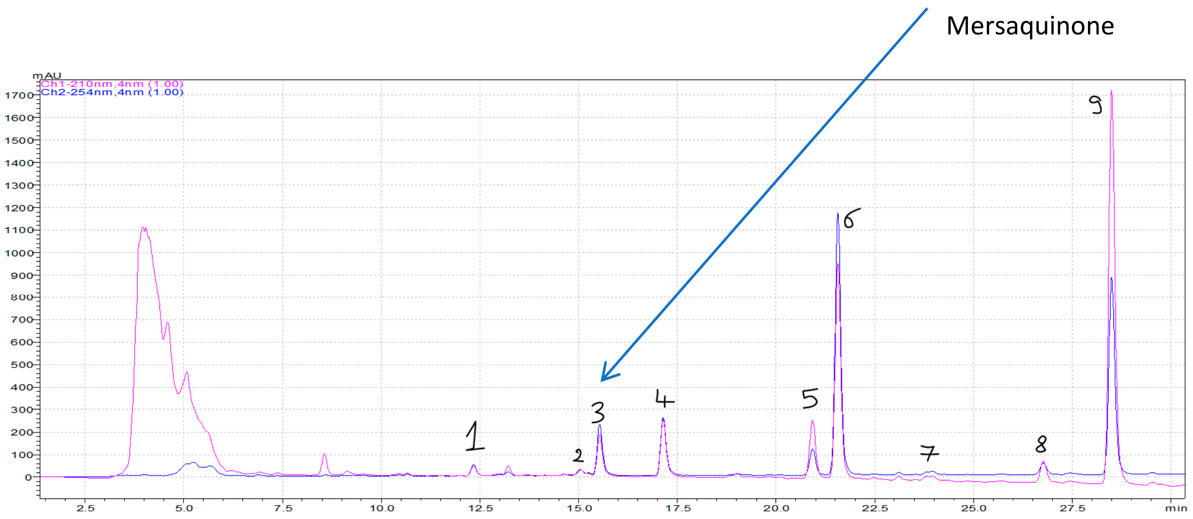
Mersaquinone
mAU
Ch1-210nm,4nm (1.00)
Ch2-254nm,4nm (1.00)
min
1
2
3
4
5
6
7
8
9

Table S1. NMR spectroscopic data for compounds **1** and **2**.

	1		2	
Position	δ_C, mult[a]	δ_H, mult (J in Hz)[b]	δ_C, mult[a]	δ_H, mult (J in Hz)[b]
1	145.2, C		164.3, C	
2	124.8, CH	7.00, br d (2.2)	108.0, CH	6.51, d (2.4)
3	162.0, C		165.1, C	
4	112.2, CH	7.45, br d (2.2)	108.1, CH	7.06, d (2.4)
4a	137.7, C		135.7, C	
5	181.8, C		180.9, C	
5a	128.0, C		127.8, C	
6	120.2, CH	7.75, s	121.5, CH	7.79, s
6a	138.8, C		139.9, C	
7	106.4, CH	6.84, br d (1.8)	111.6, CH	7.09, br d (1.9)
8	162.2, C		159.8, C	
9	104.7, CH	6.51, br d (1.8)	123.4, C	6.91, br d (1.9)
10	157.8, C		141.4, C	
10a	109.7, C		119.4, C	
11	158.0, C		166.1, C	
11a	106.1, C		106.5, C	
12	185.4, C		188.3, C	
12a	122.6, C		109.6, C	
13	23.9, CH_3	2.70, s	24.5, CH_3	2.75, s

[a]500 MHz, DMSO-d_6.[b]125 MHz, DMSO-d_6.

Compound **2** (tetracenomycin D): ^{1}H and ^{13}C NMR data, see Table S1; ESI-MS: *m/z* 337.0 [M+H]$^{+}$.

Compound **3** (resistoflavin); ^{1}H NMR (500 MHz, DMSO-d_6): δ 13.35 (OH), 12.63 (OH), 12.35 (OH), 7.29 (OH), 6.97 (1H, s, H-8), 6.52 (1H, s, H-4), 6.47 (1H, s, H-11), 2.76 (3H, s, H-14), 1.61 (3H, s, H-12), 1.54 (3H, s, H-13); ^{13}C NMR (500 MHz, DMSO-d_6): δ 203.6 (C-2), 189.2 (C-6), 183.7 (C-10), 168.4 (C-5 and C-7), 163.9 (C-3), 156.6 (C-11a), 150.1 (C-9), 149.5 (C-11c), 148.4 (C-11d), 127.7 (C-11), 121.2 (C-8), 120.5 (C-9a), 111.6 (C-6a), 108.6 (C-5a), 107.6 (C-2a), 104.4 (C-4), 62.0 (C-11b), 46.7 (C-1), 31.2 (C-12), 24.4 (C-13), 23.7 (C-14); ESI-MS: *m/z* 393.0 [M+H]$^{+}$.

Compound **4** (resitomycin); ^{1}H NMR (500MHz, DMSO-d_6): δ 1.55 (6H, s, Me-1), 2.88 (3H, s, Me-9), 6.32 (1H, s, H-4), 6.98 (1H, s, H-8), 7.24 (1H, s, H-11), 11.94 (1H, s, OH-10), 13.98 (1H, s, OH-5), 14.33 (1H, s, OH-3), 14.48 (1H, s, OH-7);^{13}C NMR (125 MHz, DMSO-d_6):δ 26.0 (Me-9), 28.8 (Me-1), 46.3 (C-1), 100.5 (C-4), 102.8 (C-2a), 106.1 (C-5a), 107.0 (C-6a), 107.3 (C-11b), 110.5 (C-11), 114.4 (C-9a), 119.6 (C-8), 129.0 (C-9b), 139.9 (C-11c), 152.4 (C-9), 152.9 (C-11a), 163.7 (C-10), 168.2 (C-7), 170.1 (C-5), 170.6 (C-3), 179.2 (C-6), 205.2 (C-2). ESI-MS: *m/z* 377.0 [M+H]$^{+}$.

Figure S2. ^{1}H NMR data of mersaquinone (**1**), 500 MHz, DMSO-d_6.

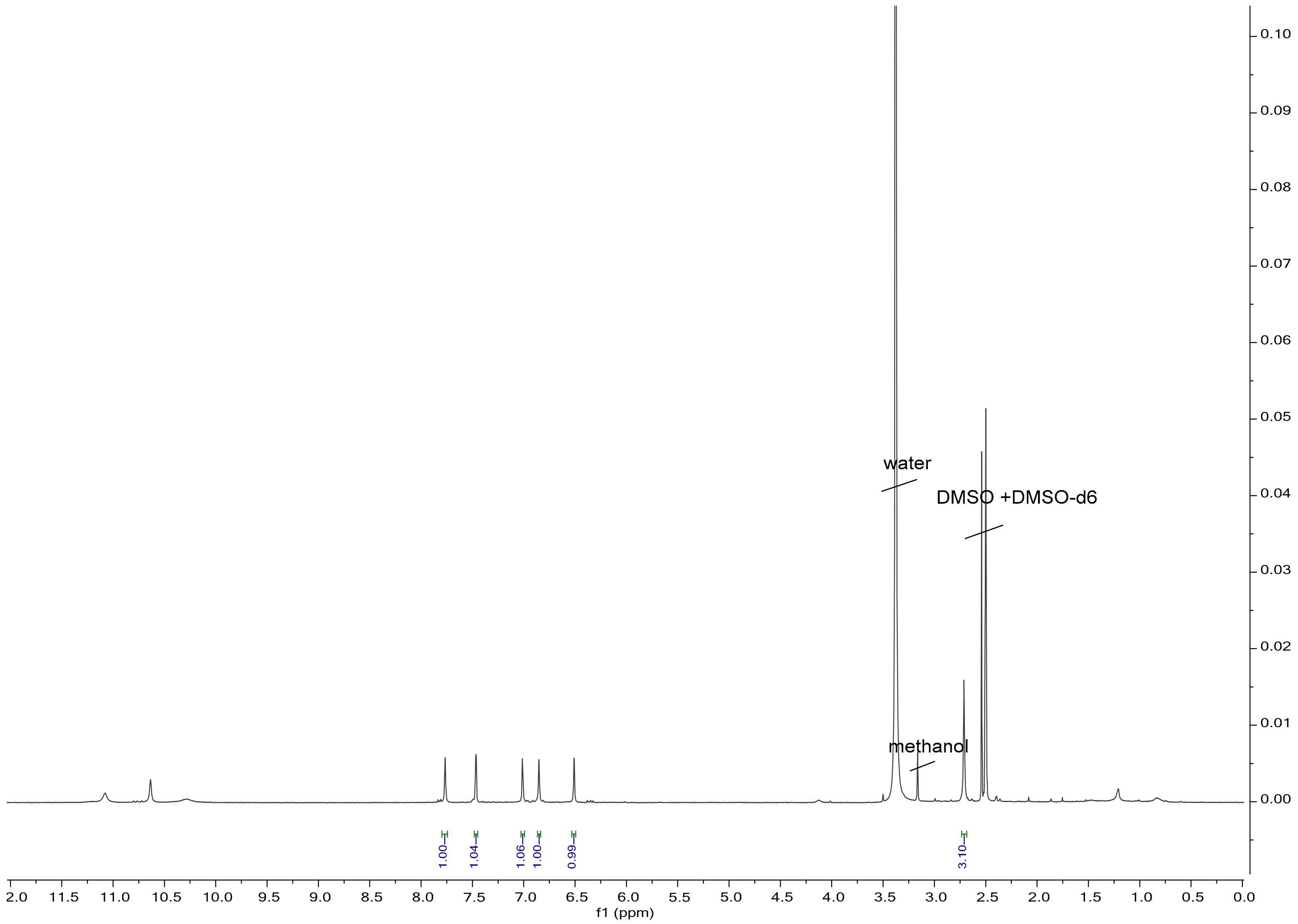

Figure S3. ^{13}C NMR data of mersaquinone (**1**), 125 MHz, DMSO-d_6.

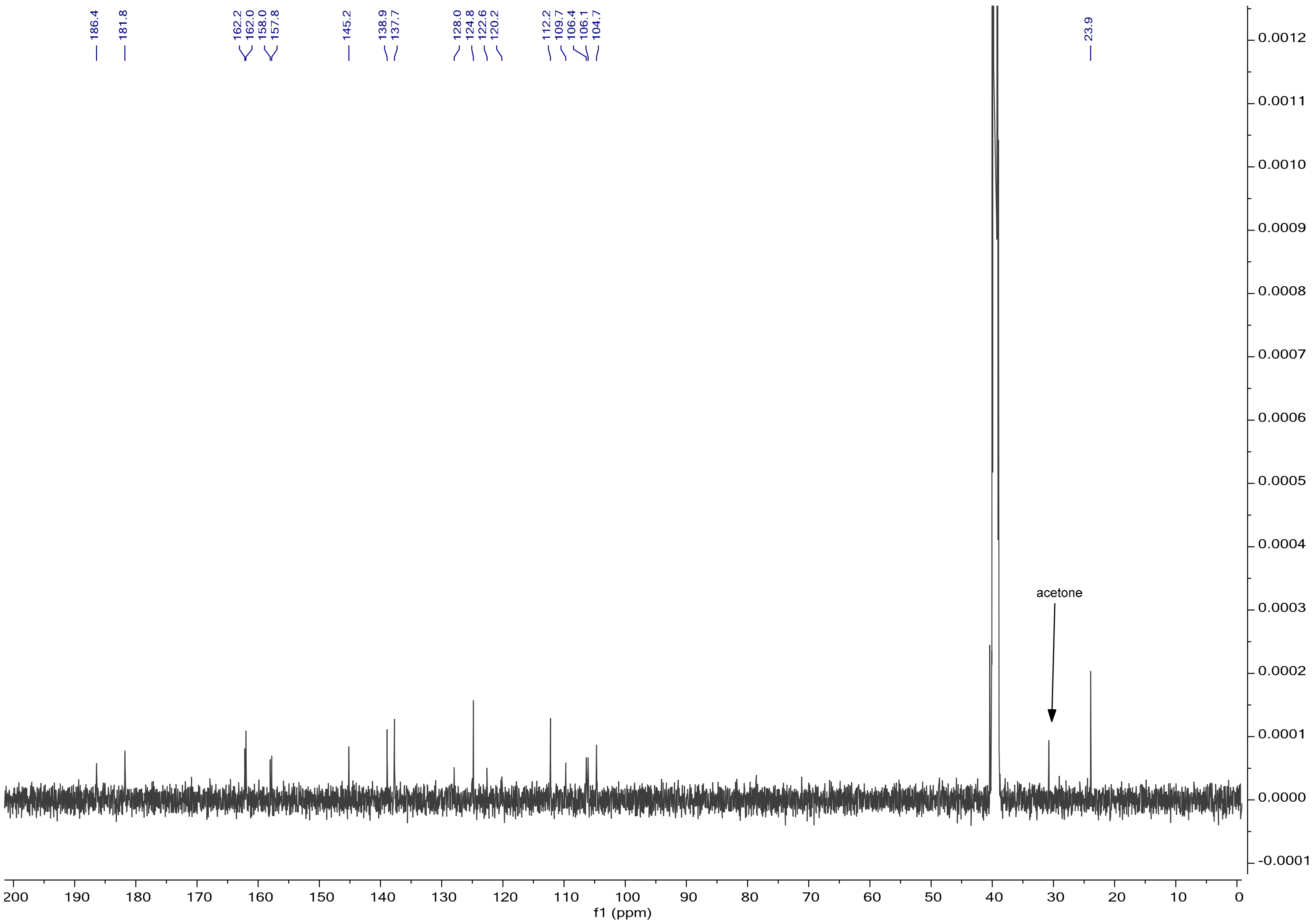

Figure S4. gHSQC NMR data of mersaquinone (**1**), 500 MHz, DMSO-d_6.

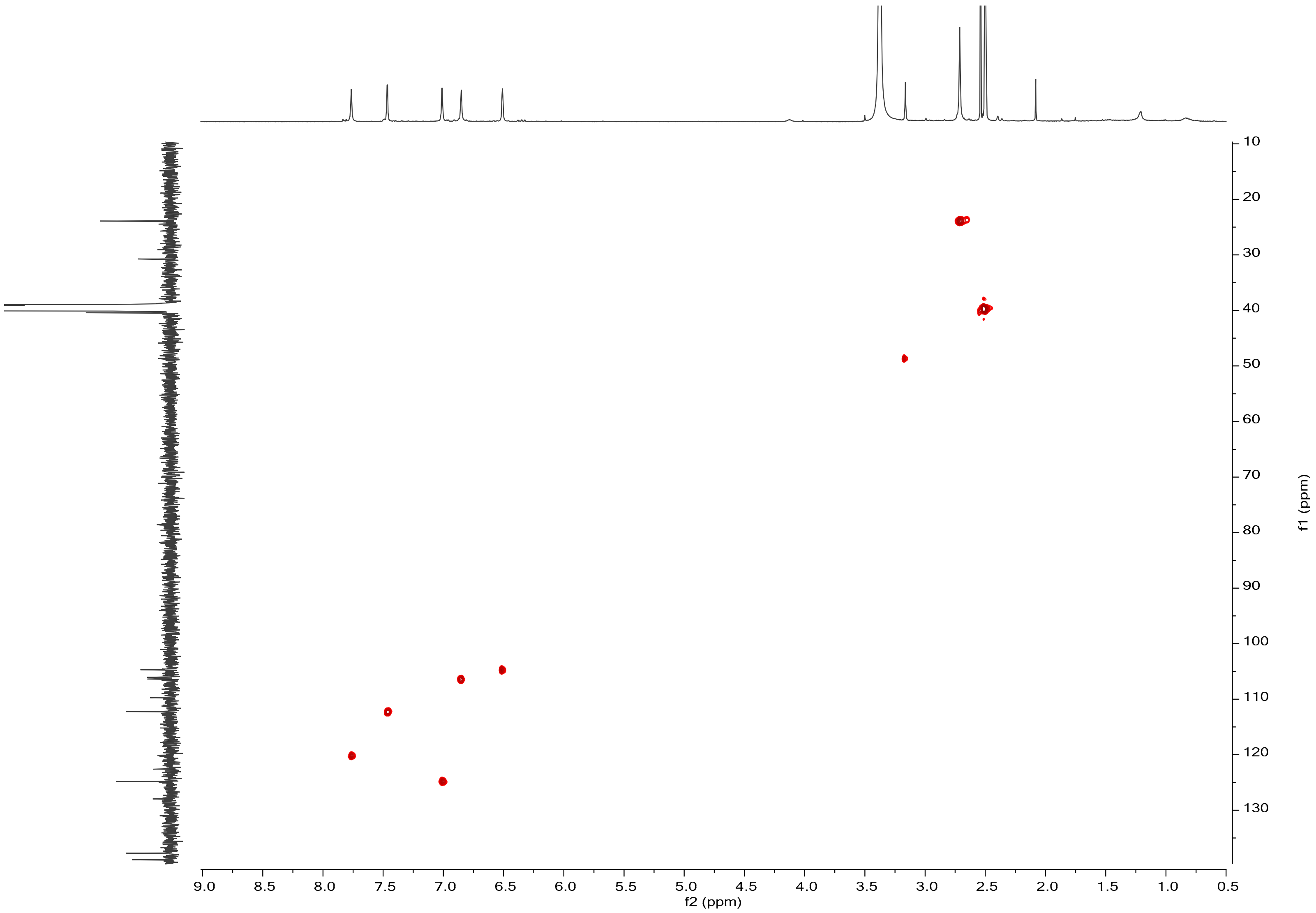

Figure S5. ^{1}H-^{1}H COSY NMR data of mersaquinone (**1**), 500 MHz, DMSO-*d*$_6$.

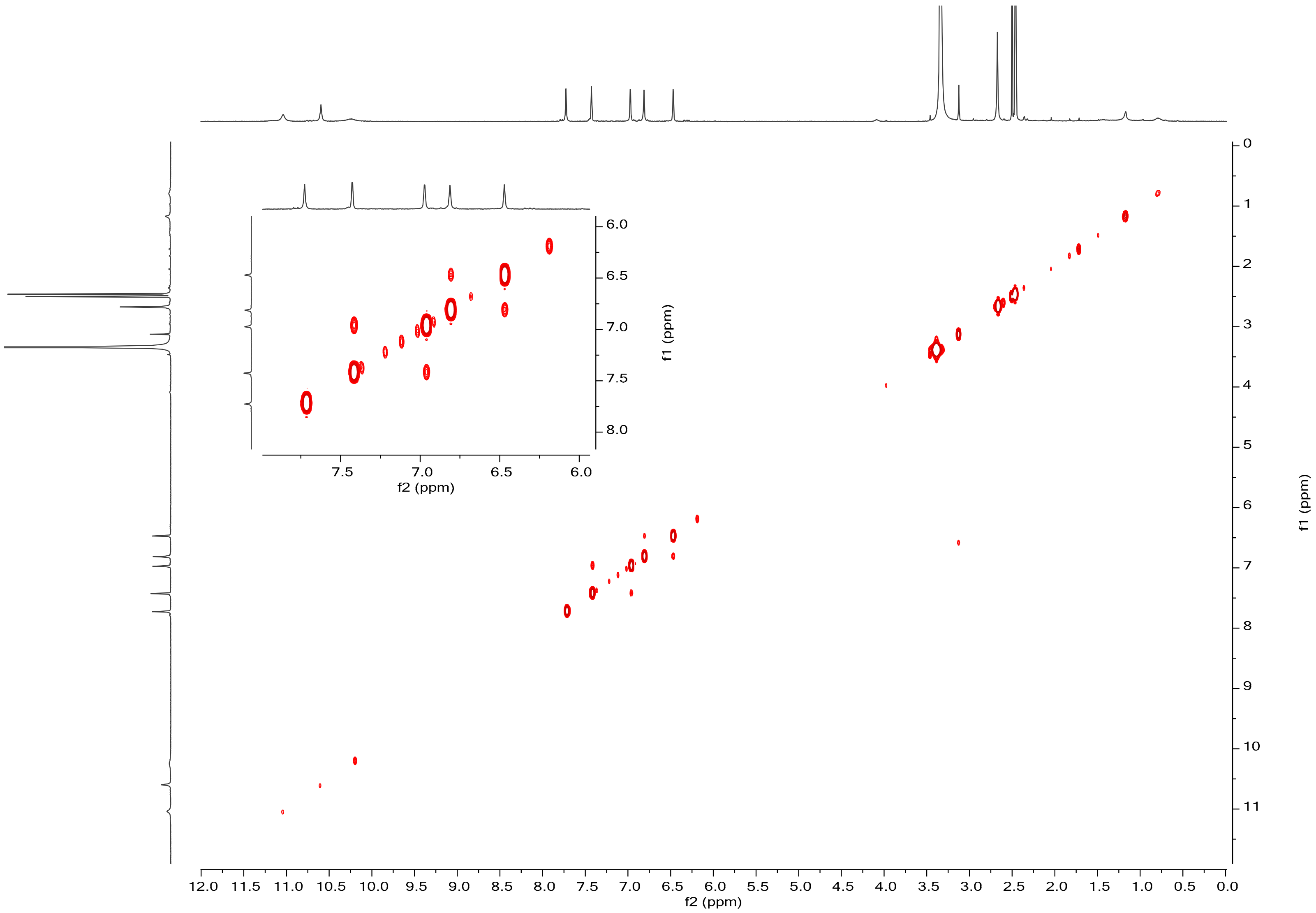

Figure S6. gHMBC NMR data of mersaquinone (**1**), 500 MHz, DMSO-d_6.

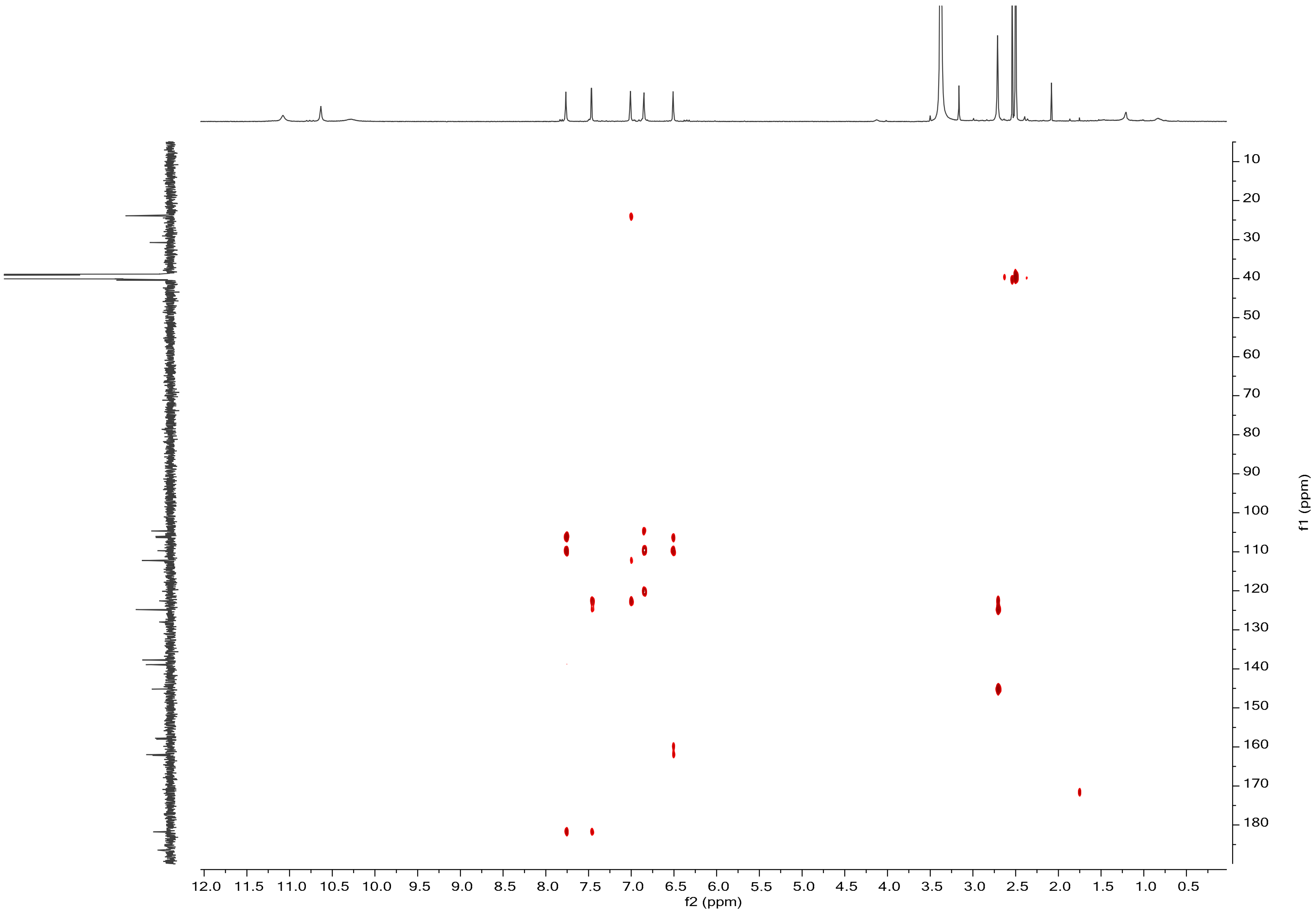

Figure S7. ^{1}H NMR data of compound **2**, 500 MHz, DMSO-d_6.

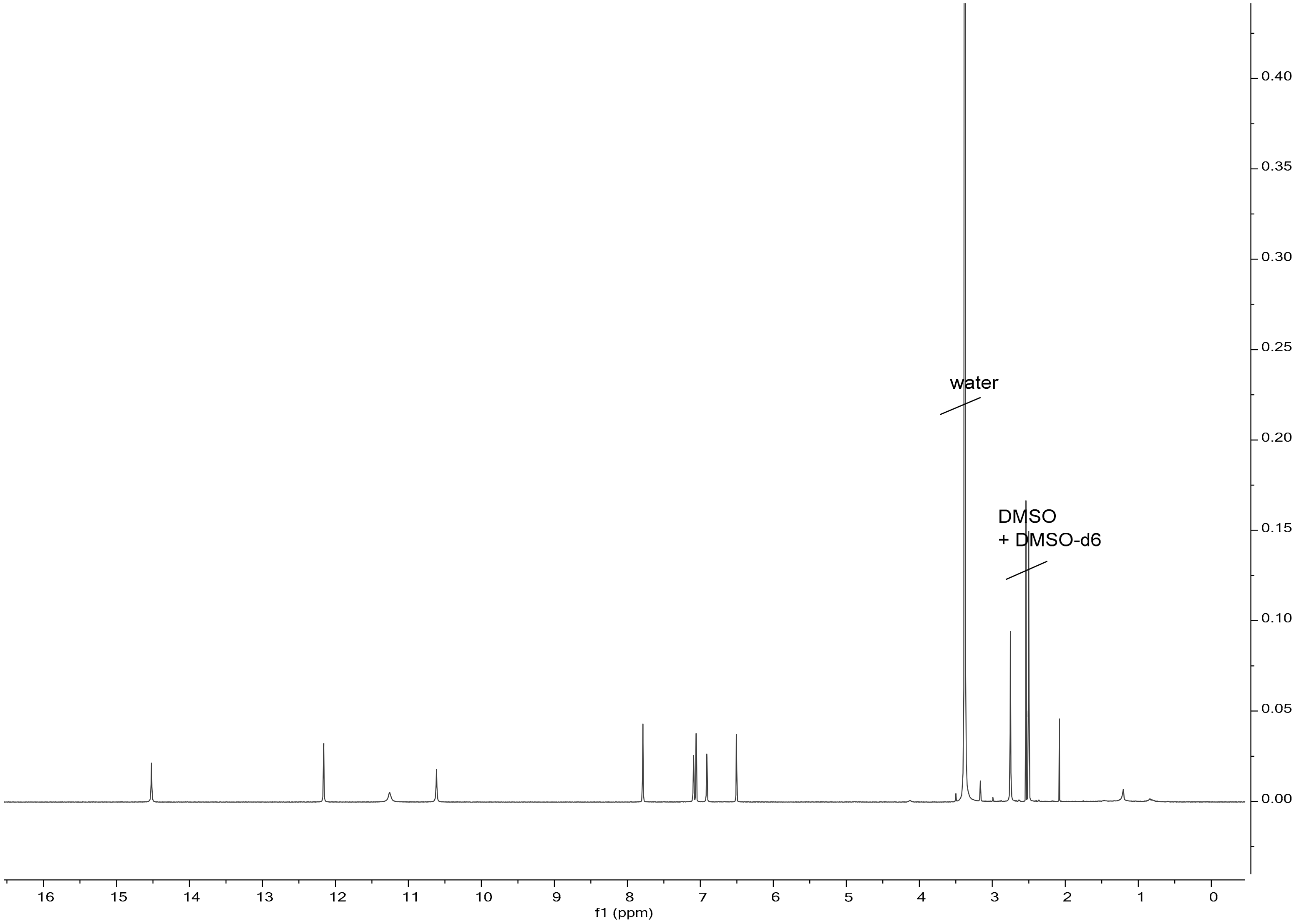

Figure S8. ^{13}C NMR data of compound **2**, 125 MHz, DMSO-d_6.

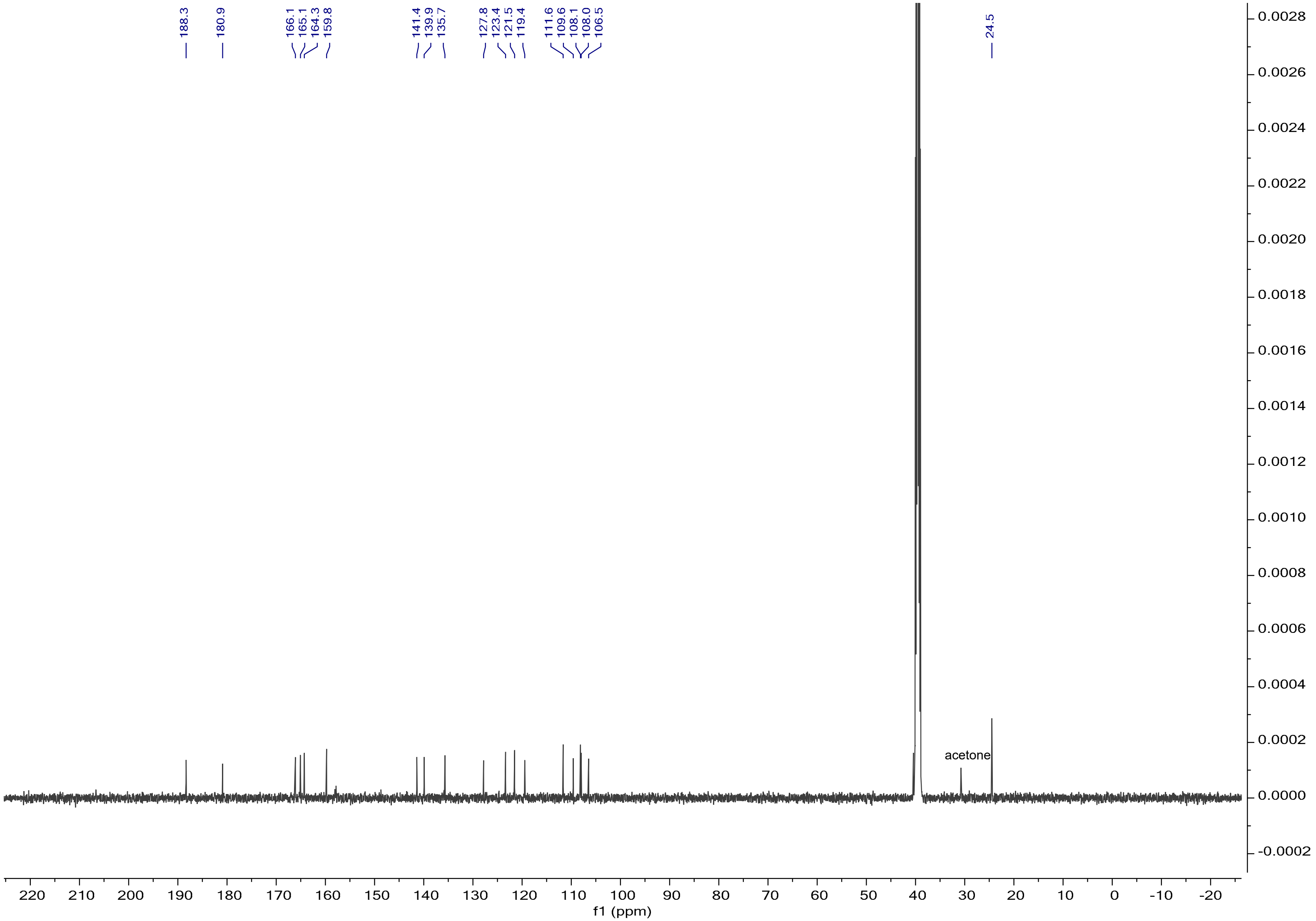

Figure S9. ^{1}H-^{1}H COSY NMR data of compound **2**, 500 MHz, DMSO-d_6.

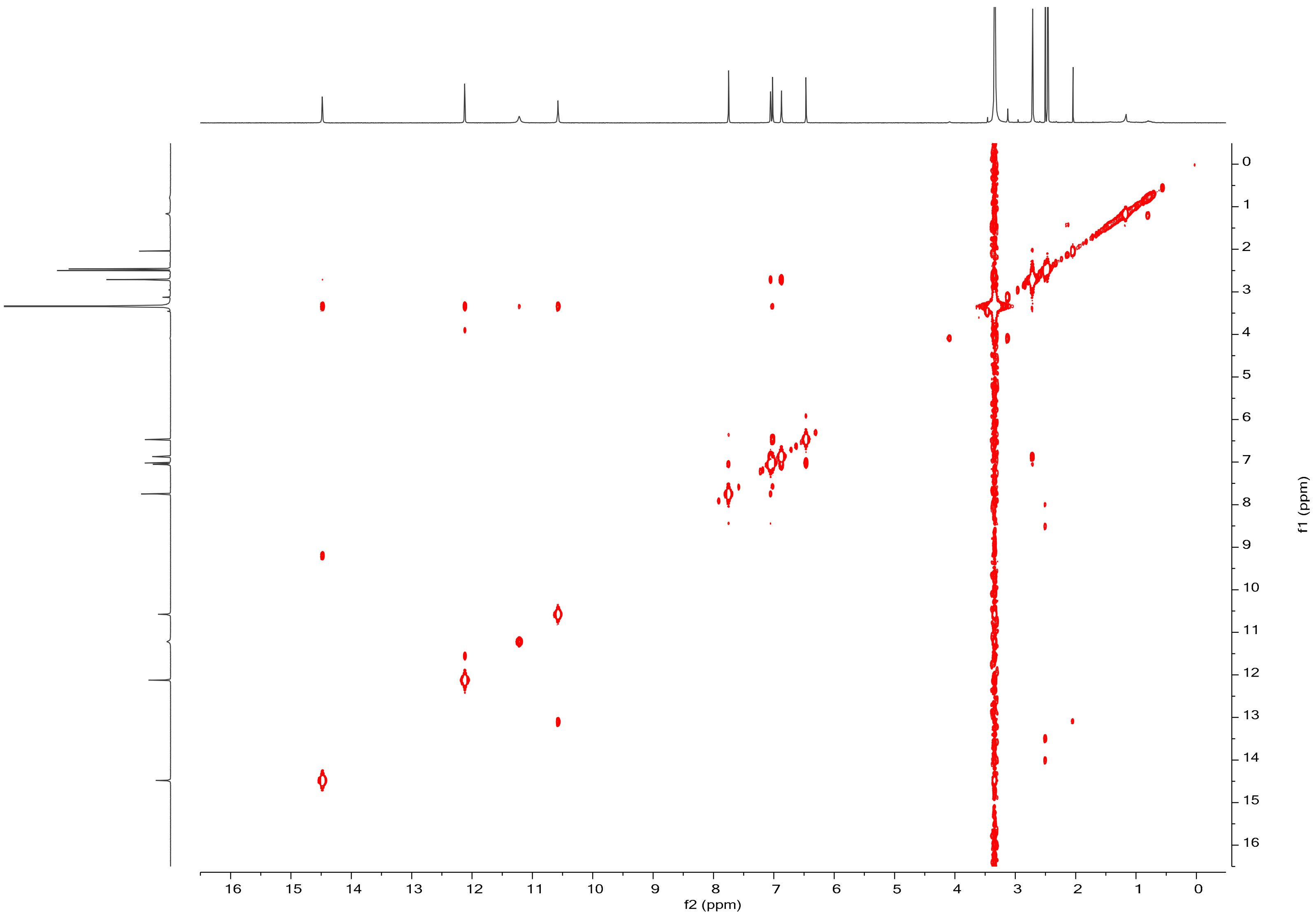

Figure S10. gHSQC NMR data of compound **2**, 500 MHz, DMSO-d_6.

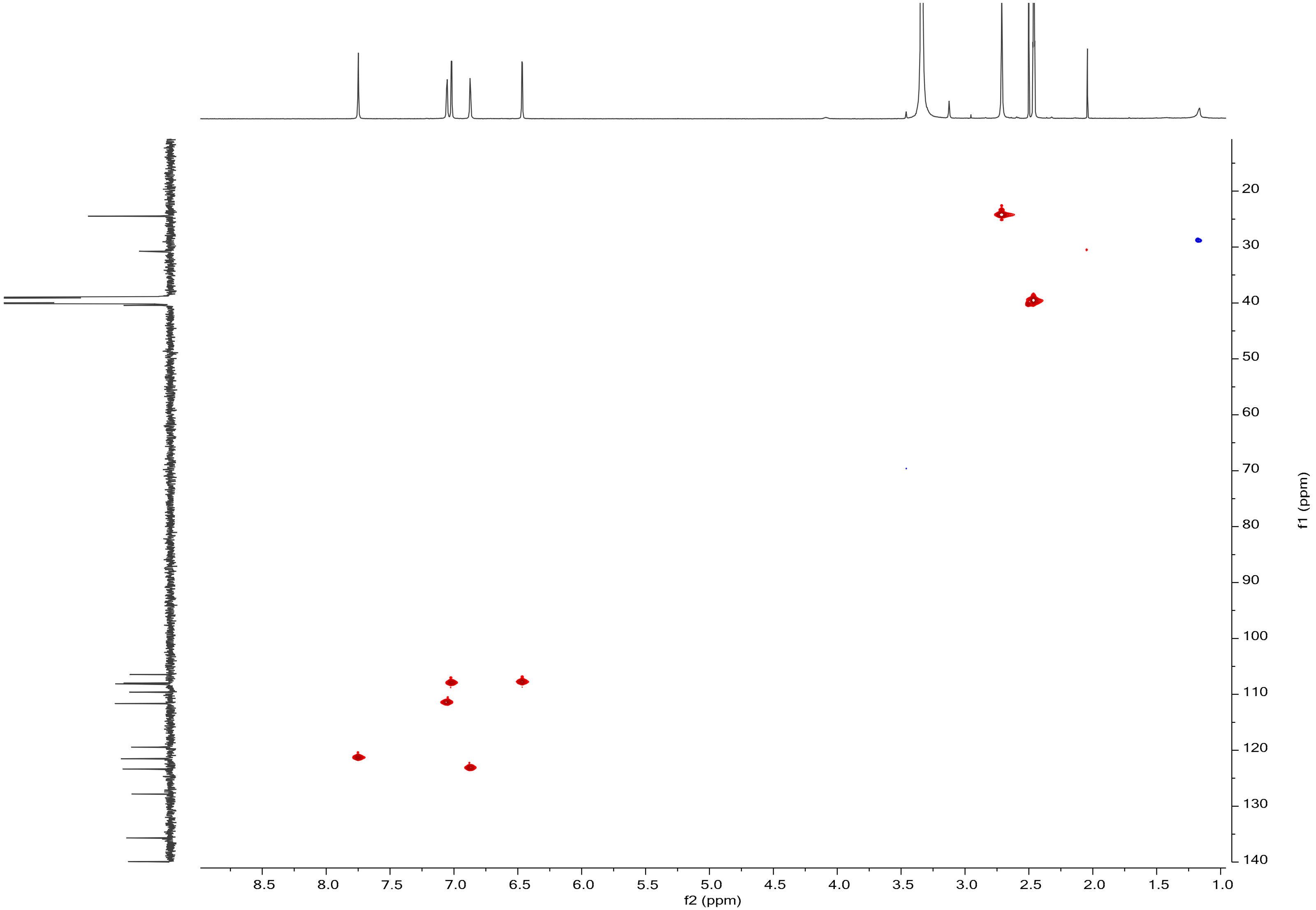

Figure S11. gHMBC NMR data of compound **2**, 500 MHz, DMSO-d_6.

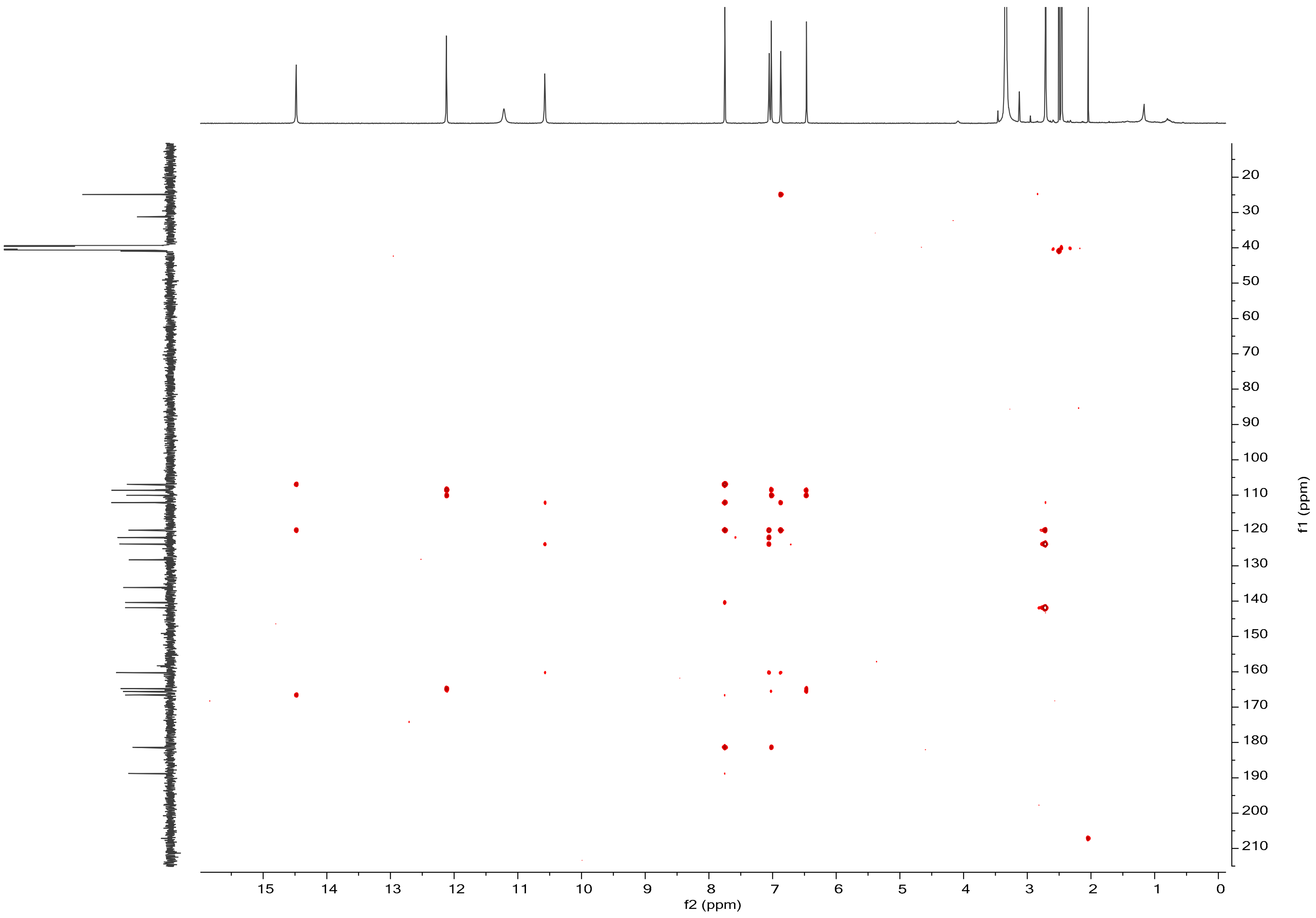

Figure S12. ^{1}H NMR data of compound **3**, 500 MHz, DMSO-d_6.

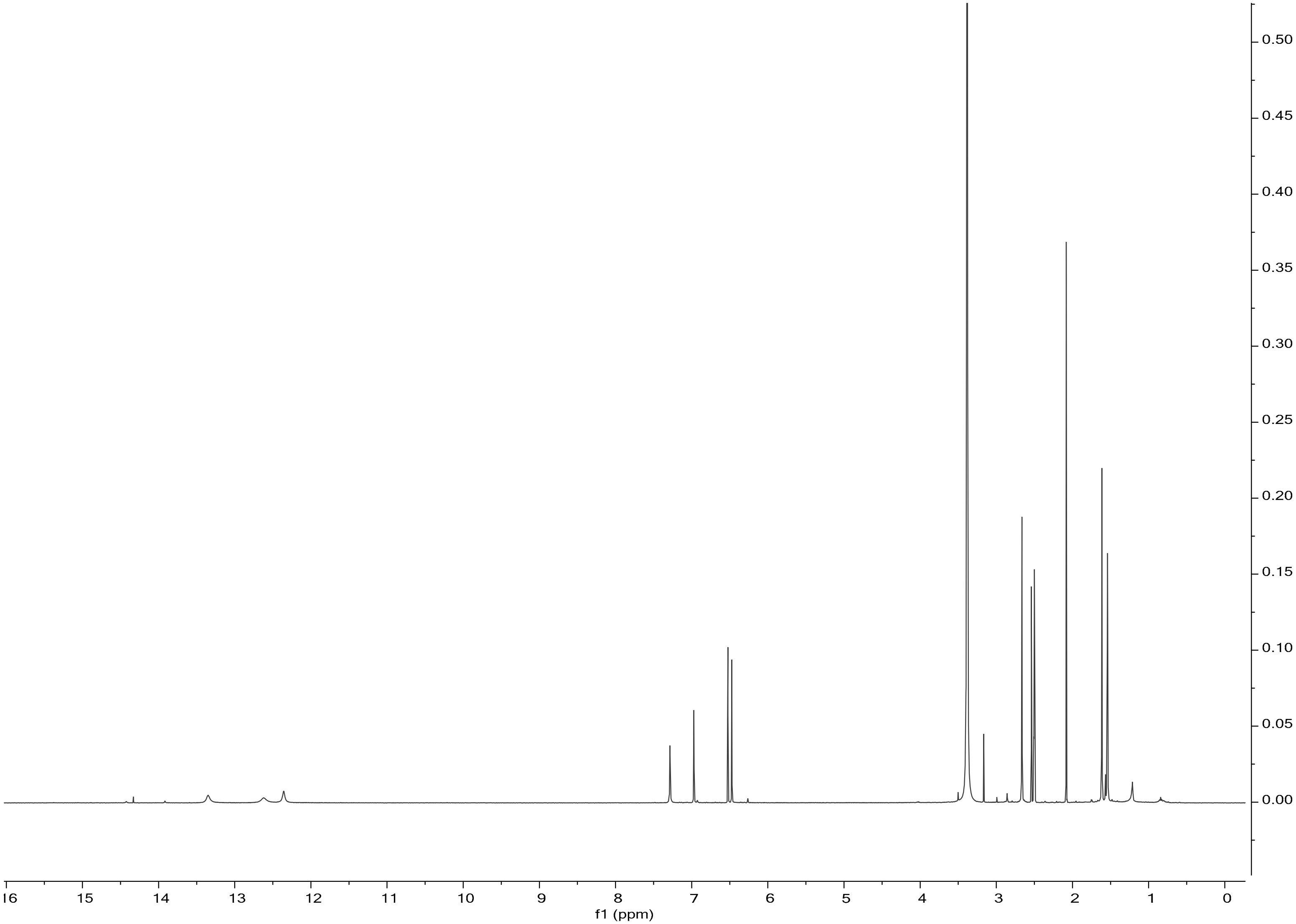

Figure S13. ^{13}C NMR data of compound **3**, 125 MHz, DMSO-d_6.

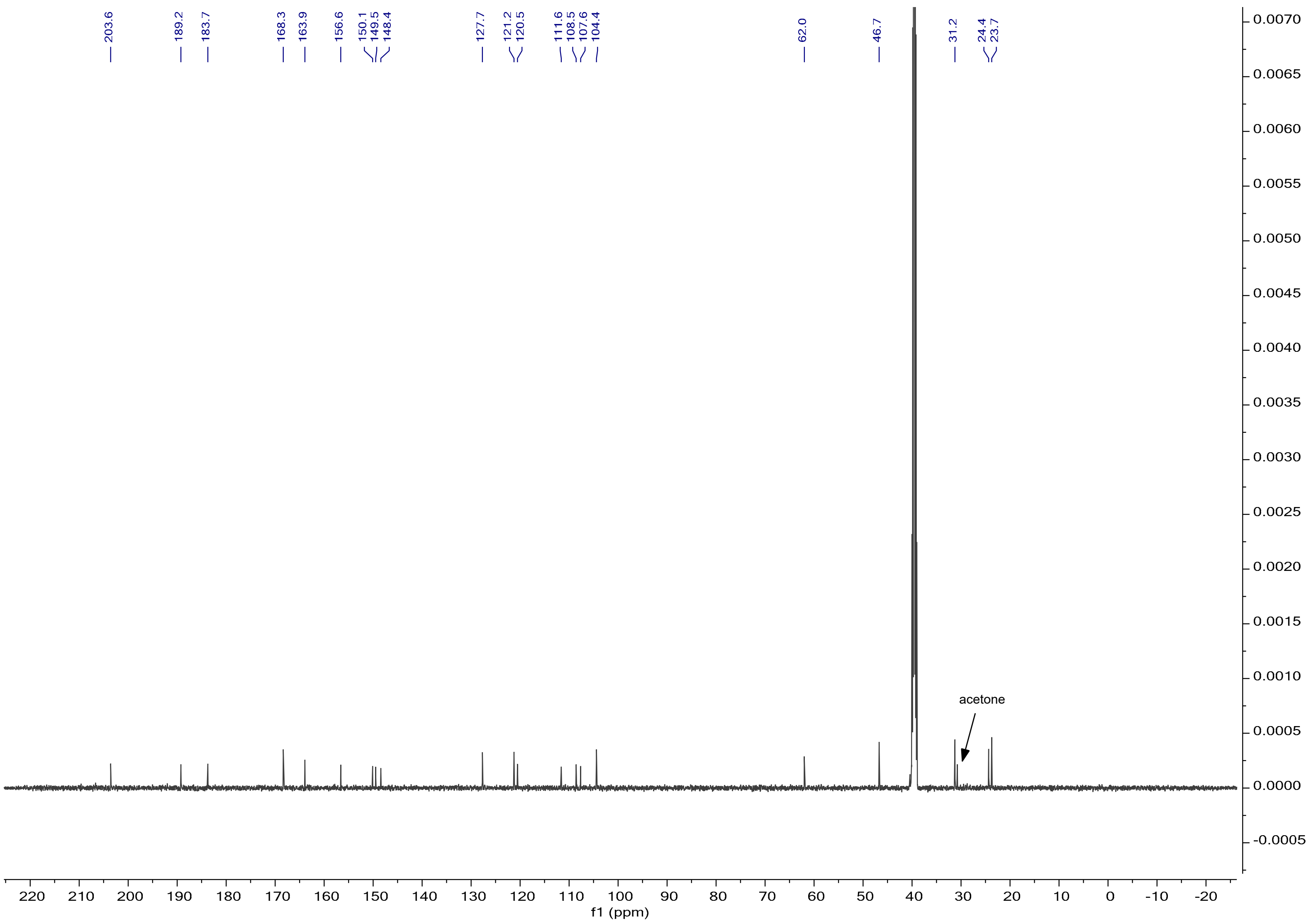

Figure S14. gHSQC NMR data of compound **3**, 500 MHz, DMSO-d_6.

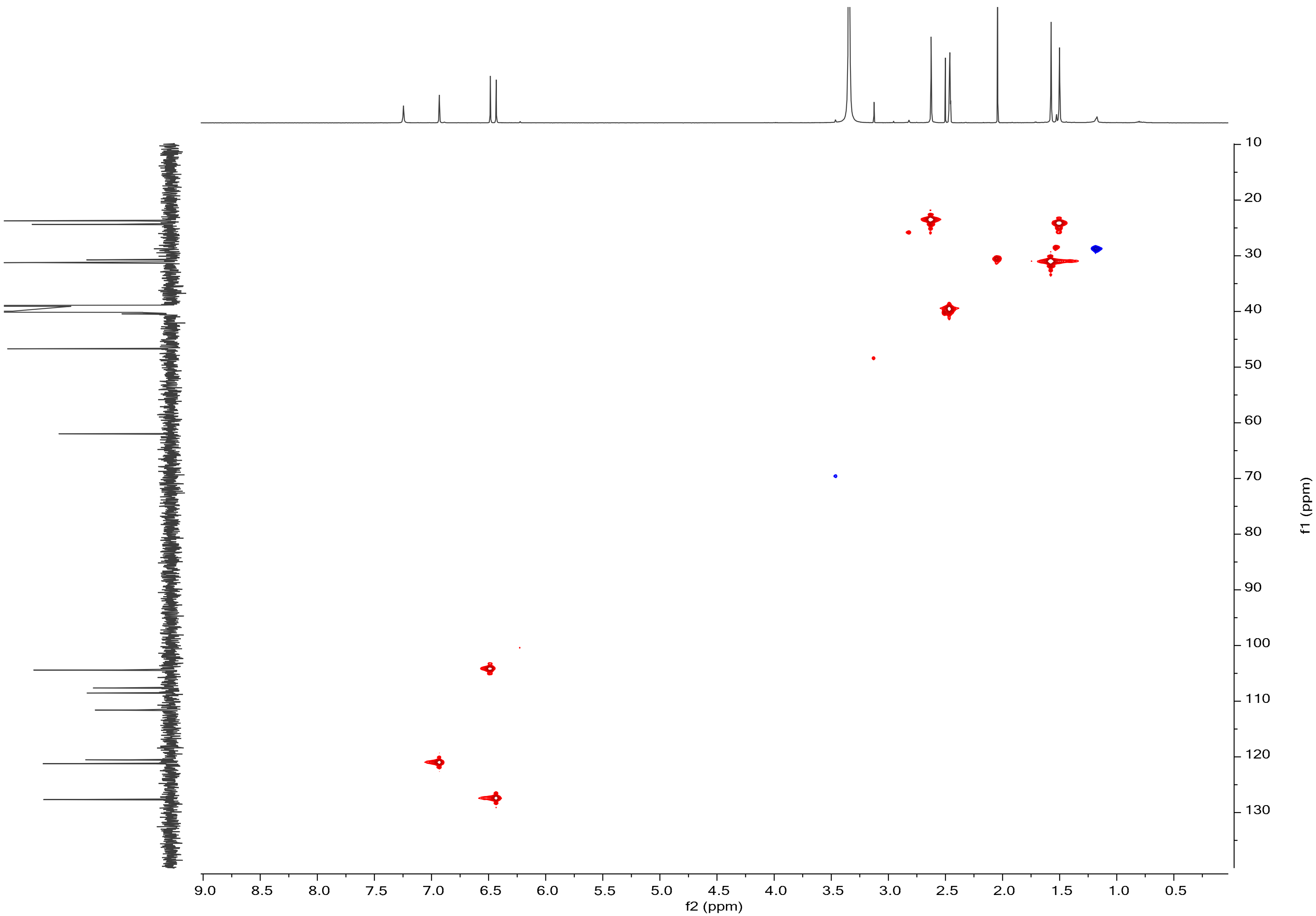

Figure S15. gHMBC NMR data of compound **3**, 500 MHz, DMSO-d_6.

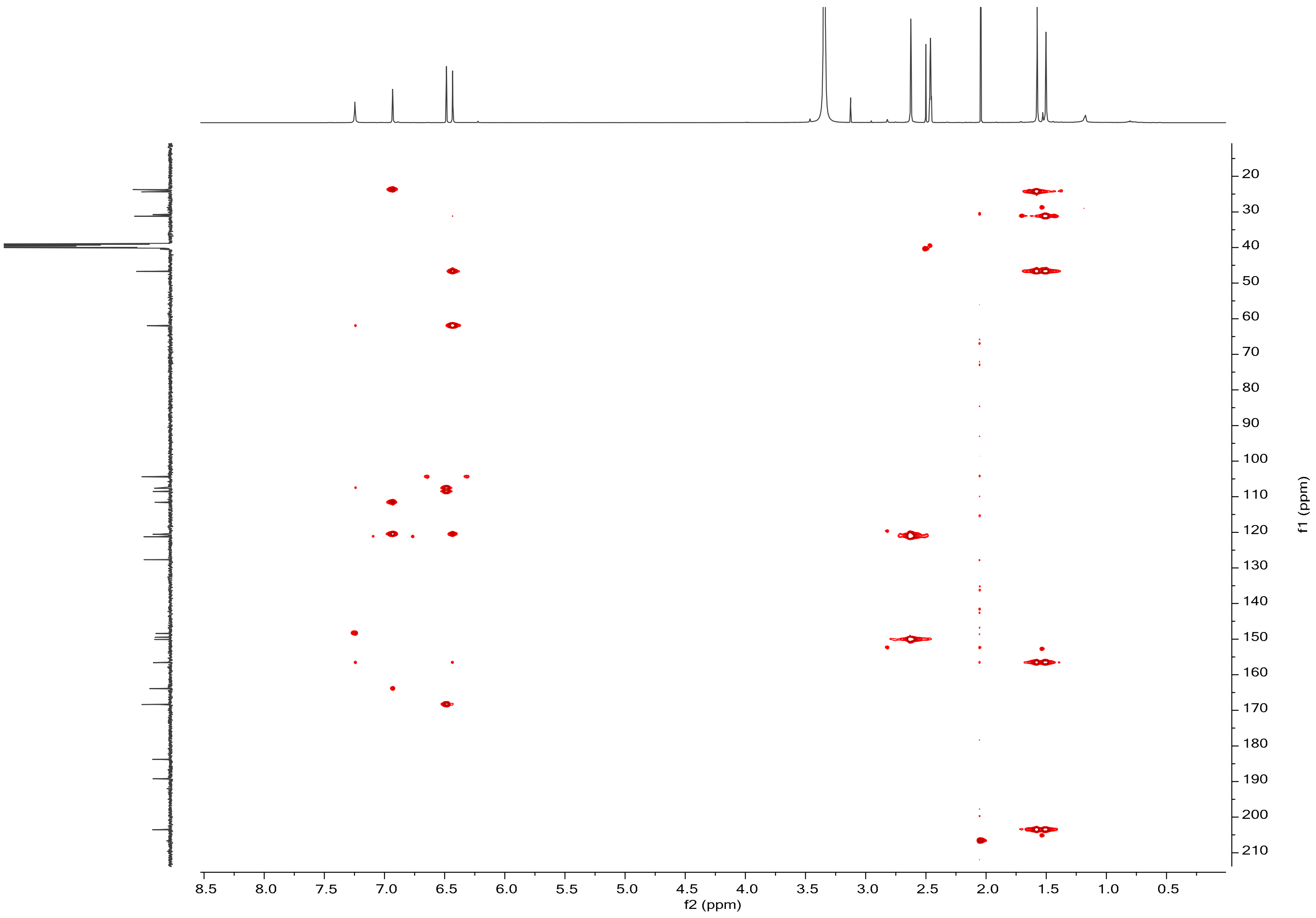

Figure S16. ^{1}H NMR data of compound **4**, 500 MHz, DMSO-d_6.

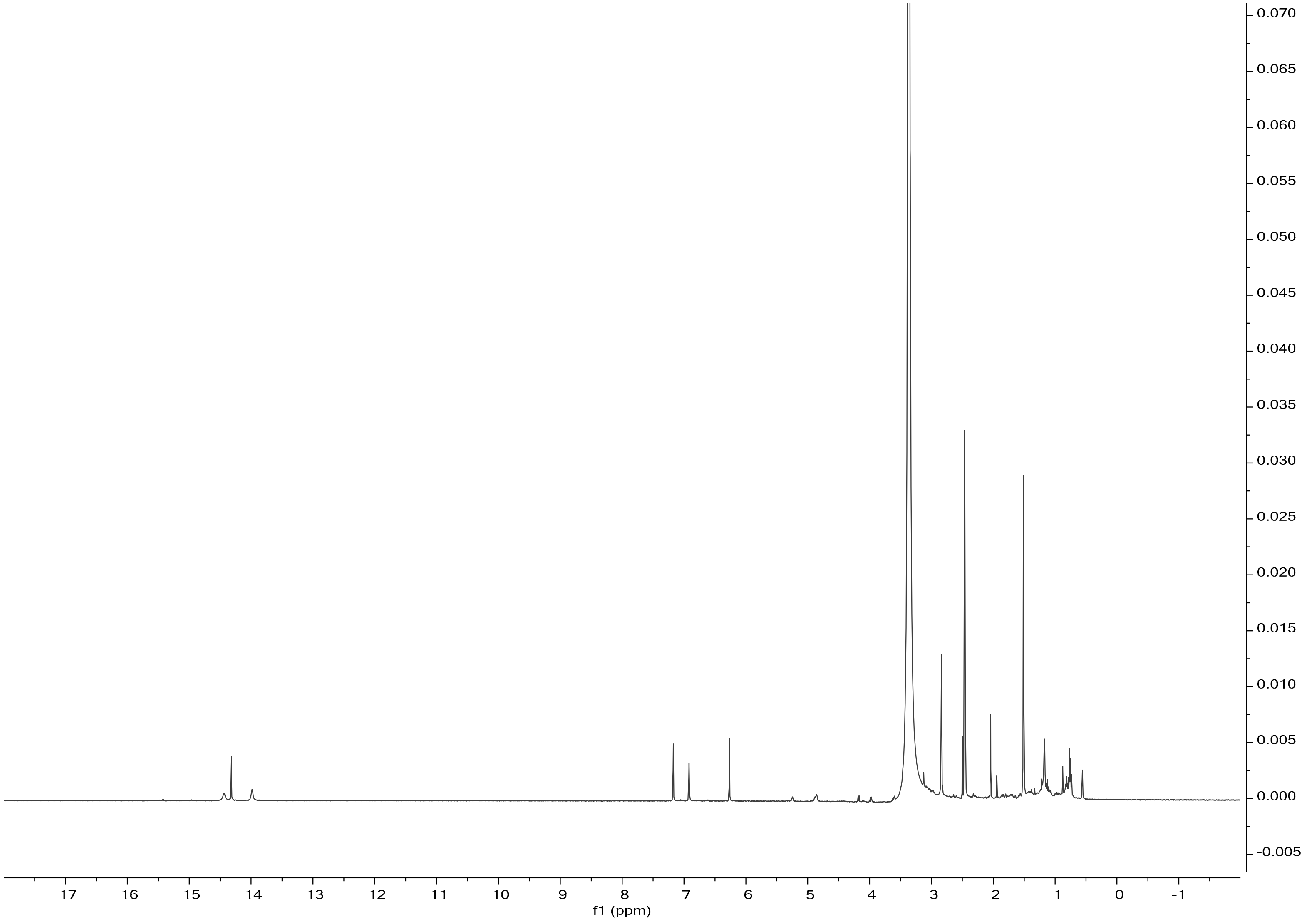

Figure S17. ^{13}C NMR data of compound **4**, 125 MHz, DMSO-d_6.

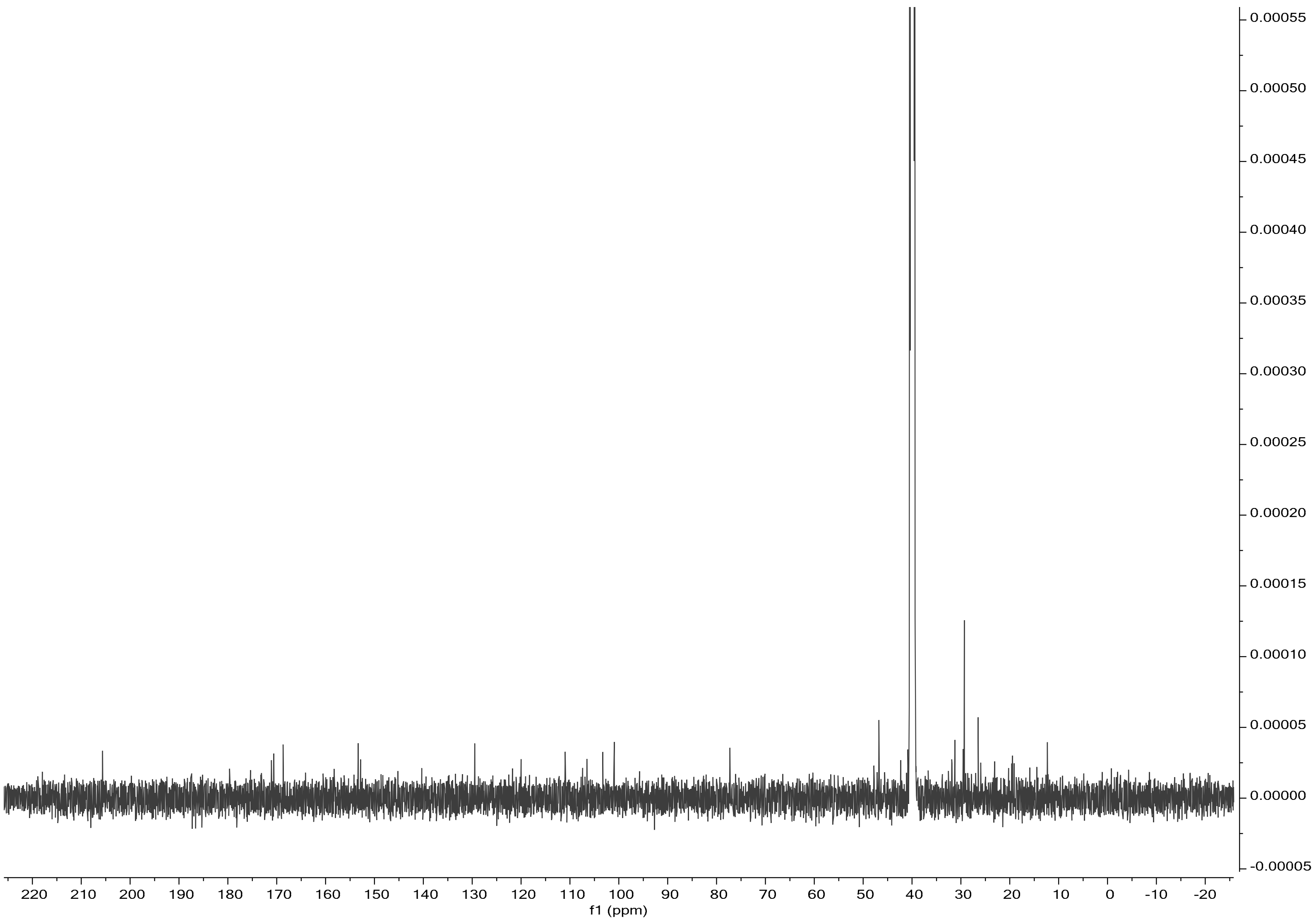

Supplement: Supplementary file 1 [file antibiotics-09-00252-s001.pdf]
